# Successful Management of Acquired von Willebrand Syndrome Associated with Monoclonal Gammopathy of Undetermined Significance After Sotorasib Treatment in a Patient with Non-Small-Cell Lung Carcinoma

**DOI:** 10.3390/hematolrep17020021

**Published:** 2025-04-16

**Authors:** Mélissa Julien, Léa Pierre, Anne-Cécile Gérout, Laurent Sattler, Olivier Feugeas, Dominique Desprez

**Affiliations:** 1 Centre de Ressources et Compétences des Maladies Hémorragiques Constitutionnelles, Hôpitaux Universitaires de Strasbourg, 67 091 Strasbourg, France; lea.pierre@chru-strasbourg.fr (L.P.); olivier.feugeas@chru-strasbourg.fr (O.F.); 2 Service de Pharmacie, Hôpitaux Universitaires de Strasbourg, 67 091 Strasbourg, France; anne-cecile.gerout@chru-strasbourg.fr; 3 Laboratoire D’hématologie, Hôpitaux Universitaires de Strasbourg, 67 091 Strasbourg, France; laurent.sattler@chru-strasbourg.fr

**Keywords:** case report, acquired von Willebrand disease, tyrosine kinase inhibitor

## Abstract

**Background:** This case report investigates the effects of sotorasib treatment in a patient with acquired von Willebrand syndrome (AVWS) associated with monoclonal gammopathy of undetermined significance (MGUS), who subsequently developed non-small-cell lung carcinoma (NSCLC) with a KRAS G12C mutation. **Case Presentation:** The patient, a 79-year-old male, presented with a prolonged history of recurrent lower gastrointestinal bleeding attributed to digestive angiodysplasia, which had persisted for over 30 years. AVWS was suspected based on a qualitative deficiency in von Willebrand factor (VWF), with abnormal results for factor VIII activity (FVIII:C), VWF antigen (VWF:Ag), and VWF ristocetin cofactor activity (VWF:Rco) (40%, 20%, and <2.4%, respectively). Further evaluation revealed the presence of an IgM kappa monoclonal spike, suggesting MGUS. In 2022, the patient was diagnosed with NSCLC harboring the KRAS G12C mutation and initiated second-line treatment with sotorasib. Notably, one year after the initiation of sotorasib therapy, the patient’s hemostasis had normalized, accompanied by significant improvements in VWF levels. VWF multimer electrophoresis demonstrated the restoration of high-molecular-weight multimers (HMWMs), and serum protein electrophoresis no longer detected MGUS. **Conclusion:** These improvements were likely attributable to the indirect effects of sotorasib on the bone marrow microenvironment. By inhibiting KRAS in stromal cells and osteoclasts, sotorasib may have disrupted the supportive niche necessary for malignant plasma cell survival, resulting in a reduction in the monoclonal spike. Unfortunately, the patient eventually succumbed to carcinogenic pleurisy.

## 1. Introduction

Acquired von Willebrand syndrome (AVWS) is a rare acquired hemorrhagic disorder, accounting for 1–3% of cases of inherited von Willebrand disease (VWD) [1]. Its clinical presentation and biological abnormalities resemble those of congenital VWD, although it typically occurs in elderly patients without a personal or family history of bleeding. AVWS most commonly develops in association with underlying diseases and may also be linked to the use of certain medications. The pathophysiology of AVWS remains complex and not fully understood, with four primary hypotheses proposed [2]. The first involves the presence of antibodies or inhibitors that bind to the von Willebrand factor (VWF), leading to the formation of immune complexes which are subsequently cleared from the circulation by the reticuloendothelial system. This mechanism is frequently observed in the context of monoclonal gammopathies and autoimmune diseases. In neoplastic conditions, the adsorption of VWF high-molecular-weight multimers (HMWMs) onto malignant cells may result in enhanced plasma clearance. The third mechanism is excessive proteolysis of HMWM, which can occur in cardiac valvopathies or in patients with a left ventricular assist device. Lastly, VWF synthesis may be reduced in hypothyroidism.

While symptomatic treatments to control life-threatening bleeding are essential, MGUS is the most frequently associated underlying disease in AVWS. AVWS can only be addressed by treating the underlying disorder. For certain etiologies of AVWS, however, clinicians are often left with limited options. In some cases, treatment options for MGUS are restricted, as it is considered a precursor to cancer, and watchful waiting is often recommended. The coexistence of AVWS with MGUS does not necessarily indicate the need for specific treatment. The patient had KRAS G12C-mutated lung cancer and was treated with sotorasib, a selective KRAS G12C inhibitor approved for second-line treatment of lung cancer. In this case report, we explore the effects of sotorasib administration in a patient with AVWS associated with MGUS, who later developed non-small-cell lung carcinoma (NSCLC).

## 2. Patient Information

The case concerns a male patient, born in 1945, who was diagnosed with AVWS at the age of 53, after experiencing repeated episodes of lower gastrointestinal bleeding due to digestive angiodysplasia. Notably, there was no personal or family history of bleeding prior to the diagnosis. Initial biological investigations revealed a qualitative deficiency of VWF, with abnormal values for factor VIII activity (FVIII:C), VWF antigen (VWF:Ag), and VWF activity using the ristocetin cofactor (VWF:Rco), respectively, 40%, 20%, and <10% (normal range 50–150%).

For the first 10 years, the clinical picture was predominantly dominated by digestive bleeding. Thus, the patient was diagnosed with Heyde syndrome, defined by the triad of severe aortic stenosis, recurrent gastrointestinal bleeding due to angiodysplasia, and AVWS. In 2005, an etiological assessment for VWD revealed a low abundance IgM kappa monoclonal spike in serum protein electrophoresis. Simultaneously, the biological diagnosis of AVWS was confirmed using advanced diagnostic techniques, including VWF multimer electrophoresis. This revealed a loss of high-molecular-weight multimers (HMWMs) and a VWF propeptide-to-VWF:Ag ratio of 3.95 (normal range < 2.4) in a patient with group O blood, supporting the acquired etiology of the patient’s VWF deficiency. Furthermore, no antibodies against VWF were detected.

Therapeutically, in the event of bleeding, the patient received iterative transfusions, but immunoglobulin infusions were unsurprisingly ineffective, given the presence of an IgM spike. The various VWF concentrates administered showed mixed efficacy, with poor recovery and a short half-life. The patient underwent multiple major interventions, including partial gastrectomy for fundic angiodysplasia, ileal resection, repair of an abdominal aortic aneurysm complicated by hemorrhagic shock, electrocoagulation, and ethmoidectomy.

Due to the onset of angina, a transcatheter aortic valve replacement (TAVR) was performed urgently in 2020, but this did not resolve the VWF deficit.

Medically, the patient developed renal failure, with a glomerular filtration rate (GFR) of 40 mL/min/1.73 m^2^, iodine-induced hyperthyroidism, treated with neomercazole, and obstructive lung disease. The monoclonal IgM spike remained consistently low.

In 2014, a pulmonary nodule was detected, but the patient refused surgery. Routine annual computed tomography scans were performed until 2022, when the patient developed dyspnea and hemoptysis. Anatomopathological examination revealed a non-small-cell lung carcinoma (NSCLC). The patient received carboplatin and paclitaxel chemotherapy in April 2022, but treatment was discontinued after one cycle due to grade 3 diarrhea, anemia, and gastrointestinal bleeding.

## 3. Therapeutic Intervention

**Figure 1:**
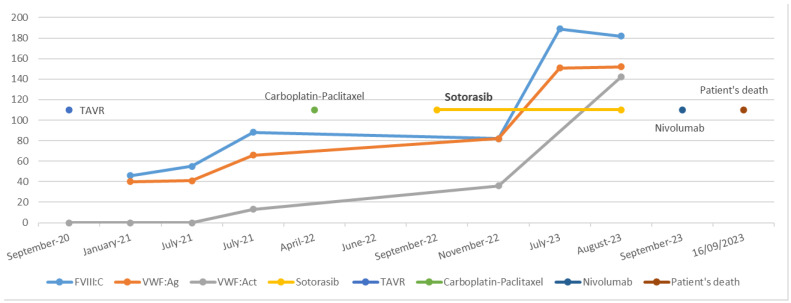
Chronological overview of therapeutic interventions and biological evolution.

In September 2022, a targeted cancer therapy with sotorasib was initiated at a dose of 960 mg (eight tablets of 120 mg), administered once daily in the morning. Two months later, in November 2022, during a follow-up appointment, the patient reported a significant reduction in hemorrhagic symptoms. Routine laboratory tests revealed changes in the profile, with FVIII:C at 82%, VWF:Ag at 82%, and VWF:Rco at 36%. However, during a follow-up consultation six months later, the patient reported no further bleeding, and hemostasis had normalized for the first time since diagnosis. Hemostasis tests remained normal on multiple occasions one year after the initiation of sotorasib. FVIII:C, VWF:Ag, and VWF:Act levels, measured using a glycoprotein Ib mutant receptor binding (VWF:GpIbM, values comparable to those obtained by VWF:Rco measurement), were 189%, 151%, and 124%, respectively (Figure 1). Multimer electrophoresis showed restoration of HMWM, and the Willebrand propeptide/antigen ratio was 0.99.

**Figure 2:**
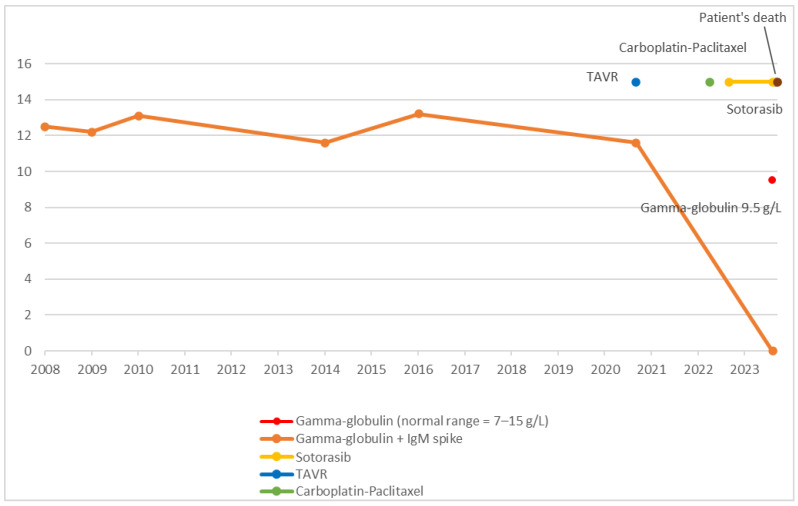
Chronological overview of therapeutic interventions and gamma-globulin evolution.

Simultaneously, protein electrophoresis, assessed on two occasions, normalized, with MGUS resolving (Cf. Figure 2).

Unfortunately, sotorasib treatment had to be discontinued in August 2023 after approximately 11 months due to tumor progression. A third-line therapy with nivolumab was initiated, but the patient received only one cycle before transitioning to palliative care. Sadly, he passed away from carcinomatous pleurisy.

## 4. Discussion

In AVWS, the primary therapeutic strategy involves identifying and treating the underlying disease. In our case, the patient presented multiple pathologies, including aortic stenosis, MGUS, and lung cancer, all of which could have contributed to the development of AVWS. Initially, the diagnosis of Heyde’s syndrome was considered. However, following TAVR, HMWMs were not restored, making the involvement of aortic stenosis in the pathogenesis of AVWS unlikely.

The association between AVWS and tumors has been reported, with VWF being absorbed by tumor cells [3]. While a potential link between AVWS and lung cancer could be hypothesized, the chronology of the patient’s medical history does not support a tumor-related etiology. AVWS was diagnosed in 1988, whereas lung cancer was identified over 30 years later. Although slow-growing tumors are a possibility, this scenario does not align with the clinical profile of the patient. Given these considerations, MGUS appears to be the most plausible causal factor.

Among lymphoproliferative disorders, MGUS is the most frequent association, representing 23% of cases [4]. MGUS does not require treatment, and severe VWF deficiency does not necessarily correlate with severe bleeding. In patients with significant hemorrhagic symptoms, anti-myeloma drugs may be considered. Case reports have shown that targeting underlying diseases associated with AVWS can normalize coagulation status [5]. Agents such as daratumumab, carfilzomib, bortezomib, cyclophosphamide, or lenalidomide have been used successfully in these patients [1,6].

Interestingly, KRAS-targeting inhibitors such as sotorasib could theoretically lead to the disappearance of a monoclonal spike in select cases, although this phenomenon has not yet been reported in the scientific literature. Three hypotheses are proposed: the first one is the expression of mutated KRAS in clonal plasma cells. If the abnormal plasma cells harbor the KRAS G12C mutation, sotorasib could suppress their proliferation, leading to the reduction in or elimination of monoclonal immunoglobulin. However, the KRAS G12C mutation was not identified in the series published by Rebmann Chigrinova [7]. A bone marrow biopsy with molecular analysis could have helped confirm the hypothesis but was not performed due to the significant risk of hemorrhage.

Another hypothesis is the disruption of key signaling pathways crucial for plasma cell survival. KRAS mutations drive cell growth and proliferation through the MAPK/ERK pathway [8,9], enhance resistance to apoptosis via the PI3K/AKT/mTOR pathway [10,11], and strengthen interactions with the bone marrow microenvironment potentially through the JAK/STAT pathway [12,13,14]. Inhibiting mutated KRAS could disrupt these pathways, leading to the apoptosis of malignant plasma cells.

The last hypothesis is an indirect effect via the bone marrow microenvironment. Even if clonal plasma cells do not directly harbor the KRAS G12C mutation, KRAS inhibition in other components of the bone marrow microenvironment, such as stromal cells or osteoclasts, may disrupt the supportive niche required for malignant plasma cell survival, potentially leading to a reduction in the monoclonal spike. This hypothesis is the most plausible explanation for the disappearance of MGUS in our case. There is no evidence that the patient’s clonal plasma cells harbored the KRAS G12C mutation, as no genetic analysis of the plasma cells was performed. Even if plasma cells were not directly affected, sotorasib may have altered the bone marrow microenvironment, disrupting signals essential for MGUS cell survival. This indirect effect could influence coagulation and VWF production regulation, unlike anti-myeloma treatments that target malignant plasma cells directly.

Given these considerations, it is important to explore other potential explanations for the observed remission. Spontaneous remission of MGUS, though rare, occurs in some cases, particularly those with low monoclonal protein levels [15]. Although spontaneous remission cannot be entirely ruled out, the timing of the disappearance of MGUS following sotorasib treatment strongly suggests a causative relationship.

## 5. Conclusions

In hematology, MGUS remains latent for many years, and no curative treatment is recommended in the absence of symptomatic disease related to malignant hematologic disorders, even when AVWS is associated. To our knowledge, this is the first reported case of AVWS associated with MGUS that resolved following treatment with a tyrosine kinase inhibitor (TKI). Further studies are needed to explore the potential role of KRAS inhibitors in modulating the bone marrow microenvironment and their implications for MGUS and AVWS management.

## Data Availability

The original contributions presented in this study are included in the article. Further inquiries can be directed to the corresponding authors.

## Abbreviations

The following abbreviations are used in this manuscript:

AVWS	Acquired von Willebrand syndrome
ENT	Ear, nose, and throat
FVIII:C	VIII factor activity
HMWMs	High-molecular-weight multimers
MGUS	Monoclonal gammopathy of undetermined significance
NSCLC	Non-small-cell lung carcinoma
TAVR	Transcatheter aortic valve replacement
VWD	von Willebrand disease
VWF	von Willebrand factor
VWF:Ag	von Willebrand factor antigen
VWF:Act	von Willebrand factor activity
VWF:Rco	von Willebrand factor activity using ristocetin cofactor
VWF:GpIbM	von Willebrand factor activity using a glycoprotein Ib mutant receptor
